# Mpox comprehensive assessment for responsive immunisation in emergency outbreaks (MpoxCARE): study protocol

**DOI:** 10.1186/s12879-026-12809-6

**Published:** 2026-02-13

**Authors:** Karishma Gokani, Herve Semukunzi, Gilbert Rukundo, Jenny Clarke, Sian E. Faustini, Jean Pierre Musabyimana, Siobhan Roche, Scott Jones, Ashley David Otter, Alex Richter, Claude Muvunyi, Jennifer Heaney, Christopher Aird Green, The MpoxCARE Study Group, Leopold Bitunguhari, Prosper Ingabire, William Iradukunda, Gerard Izuwayo, Emmanuel Kabalisa, Hin Fai Kowk, Leon Mutesa, Leonce Rukundo, Charles Simugomwa, Chloe Tanner, Jean Marie Vianney Uwimana, Philemon Uwishema

**Affiliations:** 1 School of Chemical Engineering, University of Birmingham, Birmingham, UK; 2 Department of Infectious Diseases & Tropical Medicine, NIHR/Wellcome Clinical Research Facility, University Hospitals Birmingham NHS Foundation Trust, Birmingham, UK; 3 Rwanda Biomedical Centre, Kigali, Rwanda; 4 Clinical Immunology Services, School of Infection, Inflammation and Immunology, College of Medicine and Health, University of Birmingham, Birmingham, UK; 5 Africa Centre for Excellence in Sustainable Cooling and Cold-chain (ACES), Kigali, Rwanda; 6 Emerging Pathogen Serology Group, Vaccine Development and Evaluation Centre (VDEC), UK Health Security Agency, Porton Down, UK

**Keywords:** Mpox, Outbreak, Immune diagnostic, Vaccine, Cold-chain, Seroepidemiology

## Abstract

**Background:**

Mpox disease is endemic to several regions of sub-Saharan Africa, with transmission occurring through close contact with infected animals or humans. Increasing case numbers in East Africa led the African Centre for Disease Control and Prevention to declare a Public Health Emergency of Continental Security on 13th August 2024 related to a new virus variant (clade 1b). The Mpox CARE study aims to establish clinical and epidemiology data to develop innovative immunodiagnostic tests, facilitating models for sub-clinical disease exposure, transmission and future vaccine strategies (Part A). Furthermore, we aim to evaluate and optimize vaccine cold-chain (VCC) systems to support Mpox vaccine deployment while ensuring the continued provision of childhood immunizations (Part B).

**Methods:**

This study will recruit up to 650 participants to three study groups according to Mpox exposure status and/or vaccination in Rwanda. All enrolled participants will be asked to provide demographic information, relevant medical history, up to 20mLs of venous blood and a dried blood spot capillary blood sample. For Part A of the study, samples will undergo testing for quantitative analysis of total serum antibody to Mpox specific proteins using an enzyme-linked immunosorbent assay (ELISA) and test a novel point-of-care (POC) lateral flow test to determine positive/negative qualitative serostatus. For Part B, we will collect data on VCC equipment, inventories and methods of vaccine distribution. The results of this project aim to (a) validate a blood-based ELISA assay for the detection of Mpox immunity in a target population of Rwanda, (b) generate an estimate of sero-positivity to Mpox in Rwanda, (c) evaluate the clinical performance of a novel POC lateral flow test for Mpox serostatus and (d) understand the VCC requirements for additional vaccine deployment for disease control.

**Discussion:**

Detecting antibodies for sub-clinical Mpox cases improves disease mapping and transmission patterns in endemic African regions. Novel POC immunodiagnostics can enhance community healthcare and decentralise efforts to understand Mpox seroepidemiology. Understanding VCC requirements for deployment of genetic vaccines, alongside regular vaccine provision to pregnant women, and children offers insights for management of future vaccine-preventable disease outbreaks.

**Trail registration:**

Clinicaltrials.gov trial registration number NCT06887556, registered 20th March 2025.

## Background

Mpox disease is caused by human Monkeypox virus (hMPXV), a member of the *Orthopoxvirus* (OPXV) genus [1]. Transmission occurs through close contact with infected animals’ mucosa, for example via animal bites or consumption of improperly cooked infected animals [2, 3], and between humans either through the respiratory tract or skin-to-skin contact, for example during sexual intercourse [2, 4]. Clinical infection includes coryza, fever, lymphadenopathy and the characteristic vesicular centrifugal rash [2]. Morbidity and mortality are usually secondary to bronchopneumonia, bacterial superinfection, encephalitis, keratitis leading to visual loss, or dehydration through gastrointestinal loss [3, 5]. Mpox is predominantly endemic to central and West Africa, but sporadic cases occur throughout the world [5].

Whole genome sequencing has identified two distinct clades of virus with different clinical and epidemiological features [6]. Clade I is endemic to Central Africa and the Congo Basin and is more virulent [7] than Clade II with a case fatality rate (CFR) of 10.6% [8]. Clade II is endemic to West Africa and has a CFR of approximately 4% [8]. A subset of this clade, subclade IIb lineage B.1 was responsible for the 2022 outbreak [9] when, in July 2022, the World Health Organization (WHO) declared a public health emergency of international concern (PHEIC, to May 2023) under the International Health Regulations (2005) following outbreaks in all six WHO regions [10, 11]. In 2024 case numbers in the Democratic Republic of Congo (DRC) began steadily rising [12], eventually spreading into neighbouring countries triggering the African Centre for Disease Control and Prevention to declare a Public Health Emergency of Continental Security on 13th August 2024 [13]. There are two strains of circulating virus, clade Ia (previously known as clade I) in northwestern DRC primarily affecting children, and a new strain clade Ib in northeastern DRC which has since spread to bordering countries [14]. The clade Ib strain (sub-lineage A) was first identified in South Kivu [15] and has been found to have a APOBEC3-type mutations thought to confer increased transmission between humans. Between 1 January 2024 and 2 February 2025 there have been 21,113 confirmed Mpox cases with 70 deaths [16]. The WHO released 1.45 million USD in August 2024 to fund needs-based access to smallpox vaccines in the region; however, there is currently limited global availability [17].

The clade 1b Mpox outbreak is an urgent public health emergency in need of additional scientific countermeasures to control the spread of disease and protect African communities. There are currently three vaccines recommended for use by the WHO for prevention of Mpox; (1) ACAM2000, a replication competent vaccinia virus and a second-generation smallpox vaccine (2), MVA-BN, a non-replicating modified vaccinia virus Ankara vaccine, and (3) LC16, a live attenuated minimally replicating vaccinia virus vaccine [18]. Vaccination is recommended for those at high risk of exposure, or as post-exposure prophylaxis within two weeks of significant exposure to Mpox [18]. There are no known immune correlates of protection; however, antibody titres are thought to be a potential surrogate for protection based on challenge studies with B cell depleted macaques [19]. Duration of vaccine protection is unknown. In longitudinal studies following smallpox vaccination, anti-vaccinia antibody titres measured by ELISA, as well as serum neutralising antibody titres are maintained above the presumed protective threshold for 75 years [20], and T-cell immunity also appears to be maintained for at least 20 years [20]. Data on how this translates to protection from Mpox remains limited. Development of Mpox specific immunological assays using OPXV-specific antibody profiles [21, 22]can help to distinguish between vaccine immunity, natural/hybrid immunity and cross reactive antibody from related poxvirus exposure, and improve our understanding of disease transmission and priorities for vaccination. The purpose of this study is to establish two immunodiagnostic assays to detect mpox-specific antibody in the Rwandan population; an ELISA and a blood-based lateral flow test. This study will also measure the capacity of Rwanda’s vaccine cold chain network to determine it is able to accommodate rapid mpox vaccine deployment for outbreak control purposes without disruption to routine childhood vaccination.

## Methods and analysis

### Study design and setting

**Fig. 1:**
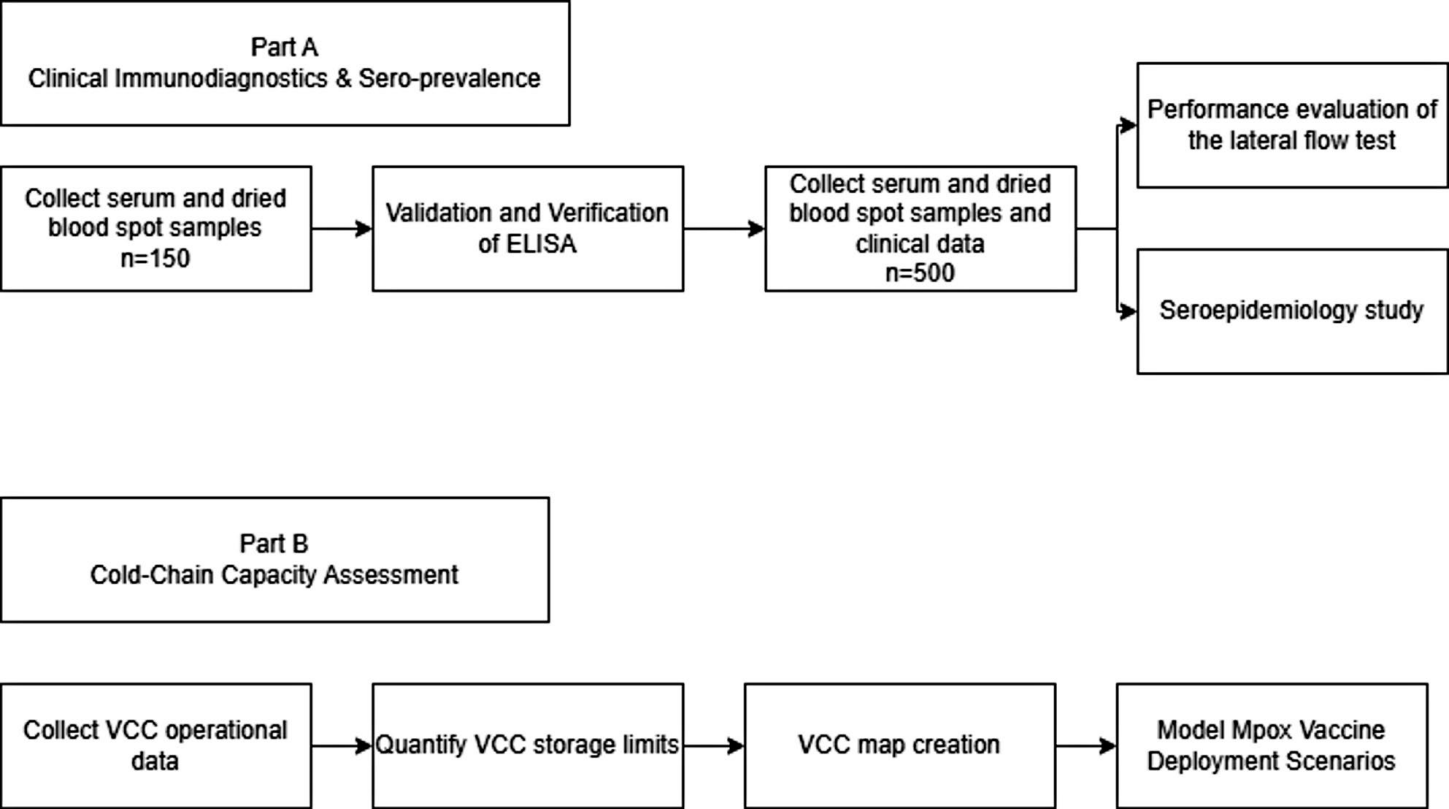
Schematic presentation of the protocol

This study consists of two parts (Fig. 1).

Part A will run in two stages, the first will validate an mpox-specific ELISA developed at University of Birmingham in a Rwandan laboratory with Rwandan samples of known clinical or vaccinated mpox status. The second stage will use this ELISA once successfully validated to perform a seroepidemiology study and evaluate the performance of a lateral flow test to identify the presence of mpox-specific antibody.

For Part B we aim to understand the vaccine cold-chain (VCC) operational needs for real-world impact of Mpox vaccines deployed for disease control.

Following rapid implementation of outbreak control measures including being the first in the region to administer mpox vaccines via a ring vaccination strategy [23], case numbers in Rwanda have remained low reaching 127 as of 5th October 2025, compared to 34,000 cases in the Democratic Republic of Congo [24]. Rwanda was selected as the study setting due to its epidemiological relevance and robust public health infrastructure. Rwanda neighbours both Uganda and DRC and we expect some overlap in background infections with cross reactivity with the ELISA, and we believe this will provide data applicable to the wider region.

The availability of meticulous vaccination and infection records enables the robust selection of well-defined mpox-case, MVA-BN vaccinated, and mpox-unexposed control groups. This structured approach, paired with the representative nature of the Rwandan Vaccine Cold Chain (managed by the Rwanda Biomedical Centre, RBC), ensures the study findings are highly applicable and generalizable to other African nations facing significant Mpox outbreaks.

## Aims and objectives

### Primary aims and objectives

Part A: The **primary clinical aim** of this study to validate novel immunodiagnostic tools to estimate Mpox sero-prevalence. Primary clinical objectives include:

- The collection of biological samples (blood) and clinical metadata from human volunteers at risk of Mpox or who have had past Mpox infection/vaccination.
- To verify a serum-based ELISA quantitative screening assay, for the detection of anti-Mpox specific IgG antibody, in a Rwandan population with equivalent sensitivity for clade 1a Mpox compared with the clade IIb that it was established for.
- To validate a prototype blood-based, binary or categorical (sero-positive/sero-negative) point-of-care lateral flow test (POC LFT) for the detection of anti-Mpox specific IgG antibody to inform further development and optimisation.

Part B: The **primary public health aim** is to understand the vaccine cold-chain readiness to deploy Mpox vaccines without detrimental effects on existing WHO EPI and other vaccine supply.

Primary public health objectives include:

- To measure the available capacity within sub-networks of the vaccine cold-chain in Rwanda to deploy Mpox vaccines into target populations.
- To model different modes of vaccine deployment scenarios for most efficient vaccine use and public health impact.

The primary endpoints of this study are

- anti-Mpox specific IgG titres as measured by the ELISA,
- Binary lateral flow test result.
- Capacity of the VCC in Rwanda as measured by number of vaccine vials stored at max capacity.

### Secondary aims and objectives

The secondary aims include how to combine findings from Part A and Part B into a further optimised vaccine response to the Mpox outbreak. We also wish to understand more about how to use serological assays for other infectious diseases, and the experiences of healthcare workers and members of the public of this work.

Secondary objectives include:

- Using data-linkage methods, biomedical data (geo-spatial case incidence data and antibody data) to map vaccine cold-chains for targeted vaccine deployment.
- Undertake exploratory immunology for insights into Mpox and other vaccine-preventable disease immunity.
- To conduct semi-structured interviews with healthcare professionals, project partners and members of the Rwandan public to understand their views and perspectives in taking part in a responsive clinical study for new disease outbreaks, the development of novel immunodiagnostic tools, and the vaccine cold-chain readiness to support public health measures.

The secondary endpoints of this study are

- Quantitative IgG titres of other vaccine preventable diseases,
- Transcripts of the focus groups.
- A map of Rwanda’s VCC.

## Recruitment and eligibility

### Recruitment

For Part A, we will use existing operational and health data records in the Rwanda Biomedical Centre (RBC) to identify, contact and invite group-specific members of the population to consider taking part in the study.

We aim to recruit volunteers to one of the following study groups:

- Group 1 (suspected exposure cohort): asymptomatic volunteers at risk of Mpox including:
  - Those who live within or adjacent to, or spend prolonged amounts of time in an epidemiologically identified region of Mpox transmission.
  - Close contacts of those with microbiologically confirmed Mpox.
- Group 2 (post-exposure/vaccinated cohort):
  - Asymptomatic volunteers who have had clinically (Table 1) or microbiologically (Table 2) confirmed Mpox infection within the last one-month (group 2a) or more than one-month ago (group 2b).
  - Those who have received a smallpox/MVA vaccine within the last one-month (group 2c) or more than one-month ago (group 2d).
- Group 3 (control cohort): asymptomatic volunteers with no known exposure to Mpox.

For Part B and the primary public health aims of the study, there will be no study volunteer recruitment or collection of personal or sensitive data. The study population here will be vaccine stock inventories in vaccine fridges. These data are already collected and available from the operational records of the Rwanda national health implementation agency, Rwanda Biomedical Centre (project partners).

### Inclusion criteria (part A)

- Healthy males and females aged between ages 5–80 years, who are able and willing to provide informed consent and will comply with the study requirements.
- Group 1 (suspected exposure cohort) only
  - Live within or adjacent to an epidemiologically identified region of Mpox transmission.
  - Close contacts of those with microbiologically confirmed Mpox.
- Group 2 (post-exposure/vaccinated cohort) only
  - Previous clinically or microbiologically confirmed Mpox or confirmed previous vaccination with a smallpox/MVA vaccine.
  - Fully recovered from Mpox infection.
- Group 3 (control cohort) only:
  - Asymptomatic with no known exposure to Mpox.

### Exclusion criteria (part A)

- Unwilling or unable to provide informed consent to take part.
- Unwilling or unable to comply with study procedures.
- History of any suspected or confirmed disorder of the immune system that, in the opinion of the investigators, might impair the results of the study.
- Have a bleeding disorder deemed significant by a member of the study team.
- Pregnant or breast-feeding females.
- Group 1 (suspected exposure cohort) only
  - Known history of Mpox infection.
  - Current symptoms consistent with Mpox.
  - Known exposure to Mpox in the last month.
- Group 2 (post-exposure/vaccinated cohort) only
  - Participants with any ongoing symptoms of Mpox, indicating incomplete recovery.
- Group 3 (control cohort only)
  - Symptoms of Mpox.
  - Known exposure to Mpox in the last month.

**Table 1:** Adapted from current WHO case definitions [23]

Clinical criteria for current or past Mpox infection
Epidemiological link to a probable or confirmed case of Mpox-21 days before symptom onset
AND
Unexplained acute rash
AND
One of the following signs or symptoms
• Headache acute onset of fever (> 38.5 °C)
• Lymphadenopathy (swollen lymph nodes)
• Myalgia (muscle pain/body aches)
• Back pain
• Asthenia (profound weakness)
AND
The following common causes of acute rash do not explain the clinical picture: varicella zoster, herpes zoster, measles, herpes simplex, bacterial skin infections, disseminated gonococcus infection, primary or secondary syphilis, chancroid, lymphogranuloma venereum, granuloma inguinale, molluscum contagiosum, allergic reaction (e.g. to plants); and any other locally relevant common causes of papular or vesicular rash.

**Table 2:** WHO guidance for laboratory testing for hMPXV virus [24]

Microbiological criteria for current or past Mpox infection
• Nucleic acid amplification testing (NAAT) using real time or conventional hMPXV (preferable) or OPXV PCR assay on either:
o Swabs from the surface of a lesion
o Swabs from exudate from a lesion
o Roofs from more than one lesion
o Lesion crusts
• IgM detection from recent acutely ill patients
• Seroconversion of IgG in paired serum samples, collected at least 21 days apart, with the first serum sample collected during the first week of illness

### Study interventions

#### Part A

All participants will provide informed consent using the latest Rwanda National Ethics Committee (RNEC) approved combined Study Information Booklet and Informed Consent Form (SIB/ICF) prior to commencement of any study procedures. Once informed written consent is obtained, the baseline assessments will be collected that include age, sex, region of residency, relevant medical history and national health number. A participant is enrolled at the point of sample provision. No formal follow-up of participants is planned as part of the study.

#### Safety

The study has minimal risk to participants. Phlebotomy carries a small risk of transient bruising and discomfort.

#### Participant samples

Each participant will be asked to provide a peripheral blood sample of up to 20mLs and a dried blood spot sample. Samples will be collected according to local standard operating procedures (SOPs).

#### Laboratory analysis

All laboratory procedures will be undertaken at the National Reference Lab in Kigali. Blood samples will undergo treatment to neutralise all infective agents and the removal of all human cells and genetic material before application to antibody-based assays in accordance with local SOPs.

All samples will undergo testing Mpox-specific IgG antibodies. The performance characteristics of the ELISA have been validated by the University of Birmingham and the United Kingdom Health Security Agency (UKHSA) during development of this ELISA. This ELISA will initially be validated and verified for use from known and presumed positive/negative study participants in Rwanda. This ELISA will then be employed as the reference standard against which performance of the prototype POC LFT will be measured.

### Sample storage

All samples will undergo pseudo-anonymisation at the study site before processing at laboratory facilities in accordance with SOPs. Participants will be given the option whether their samples can then be used anonymously for other vaccine research projects. They will also be asked whether anonymised samples could be used by commercial companies to develop other immunodiagnostic tests.

### Acceptability and feasibility assessment

A subsection of study participants will be invited to take part in semi-structured interviews/focus groups to receive feedback on the perceptions of antibody testing and disease outbreak management.

The following questions will be used as prompts to encourage full feedback from participants:

- Question 1: In relation to engagement with the public when there is a disease outbreak, what do you feel works well, needs improvement, and how would you like to see this develop further?
- Question 2: In relation to taking biological samples to develop immunodiagnostics, what do you feel works well, needs improvement, and how would you like to see this develop further?
- Question 3: In relation to preparing to deploy additional vaccines for a disease outbreak, what do you feel works well, needs improvement, and how would you like to see this develop further?

### Sample size

#### Part A

The first stage of part A will involve validation of the University of Birmingham-developed multiplex ELISA evaluated on UK samples by University of Birmingham and UKHSA following Mpox infection with clade II virus and/or vaccination [25], using *N* = 50 samples each from groups 2b, 2d and group 3. The ELISA uses several Mpox proteins and shows excellent concordance with a multiplex bead assay developed by the UKHSA [25] on UK samples, with high sensitivity/specificity for identifying seropositivity. The performance of the ELISA will be evaluated on Rwandan participants with known status to evaluate the effect of factors such as ethnicity, nutritional status, chronic viral infection (e.g. HIV or hepatitis B) and pathogen factors such as clade Ib virus (the cause of the current outbreak) on test performance. The sub-group specific recruitment targets have therefore been expanded by 50% to accommodate potential confounders and improve the stability of estimates applied to this component of the study and the next steps in validation and assessment of clinical accuracy of novel POC LFTs. Once the performance of the Mpox ELISA is confirmed in serum, alternative sample matrices including plasma and dried blood spot (DBS) will be compared to IgG titres measured in serum in paired samples.

There is a lack of data on the current sero-prevalence of Mpox-specific immunity, which this study aims to address, meaning statistically driven power calculations are challenging. Sample sizes have been calculated according to Clinical & Laboratory Standards Institute standards and consistency with the design, setup and verification protocols used for new infectious diseases targets.

The sample size calculation has been derived from the assumption of 20% seroprevalence in the intended target-use population (high-risk individuals with no known past infection/vaccination), plus added assumptions of a 5% margin of error and a 10% allowance for the withdrawal of consent from any participants, missing data, and assay failures/invalid test results.

In total, *N* = 500 is required for this part of the study from the following study groups:

- Group 1 (suspected exposure cohort): *n* = 330.
- Group 2a (infection within one-month of sampling): *n* = 10.
- Group 2b (infection over one-month before sampling): *n* = 50.
- Group 2c (vaccination within one-month of sampling): *n* = 10.
- Group 2d (vaccination over one-month before sampling): *n* = 50.
- Group 3 (no known past exposure to infection or vaccination): *n* = 50.

This sample size is sufficient for stability of estimates on POC LFT performance that will be deployed in parallel to the same study volunteers at the time of sampling.

#### Part B

For Part B and the primary public health aims of the project, we intend to collect vaccine stock inventories at different levels of the VCC using a study-specific mobile application.

Data collected from this study will also use publicly available metadata (such as the 2022 Rwanda Population and Housing Census), operational data collected as part of routine service delivery (such as road milage, RBC national VCC audit data), and data derived from other related research projects.

### Statistical analysis plan

#### Part A

The analyses for this study will be primarily descriptive in purpose (counts/proportions, or geometric mean titre where appropriate) and will not include any hypothesis testing. Comparative analyses (chi-squared/Fisher-exact or paired/unpaired t-test where appropriate) and the presentation of p values will represent post-hoc analyses.

ELISA performance will be assessed by a receiver operating characteristic (ROC) curve and area under the curve analysed to evaluate the ability of the ELISA to distinguish those presumed seropositive (vaccination and infection) from seronegative (no known infection or vaccination). The best cut-off for identifying serostatus will be selected using this method based off the highest sensitivity and specificity.

The Mpox POC LFT performance will be evaluated versus the ELISA and characterised by positive/negative concordance, correlation, test sensitivity, specificity, positive predictive value (PPV), negative predictive value (NPV) and likelihood ratios. The views and perspectives gained from the semi structured interviews will be analysed qualitatively on a small subset of participants (*n* = 30).

#### Part B

This data will be analysed descriptively using counts of vaccines by number of doses and number of vials and capacity for these at each point of the vaccine cold chain. This data will be used to generate a map of Rwanda’s VCC network.

### Data management plan

For Part A and primary clinical aims of the study:

Confidential records used to identify, contact and invite group-specific potential volunteers will be accessed by members of the RBC team only. From here, study participant data will undergo pseudo-anonymisation at source and all study documentation and biological samples will refer to a unique study number. The link between participant identification and study identification will be stored separately by RBC and not shared with other partners, except for meeting the needs of regulatory authorities and sponsor monitoring. These data are collected and stored onto a REDCap clinical database on encrypted RBC servers. Access to the database will be restricted to authorised members of the study team under password-controlled methods. Research data generated by the study remains the property of RBC and will be shared with the University of Birmingham and other invited partners to support the delivery of the study aims and objectives.

For Part B and primary public health aims of the study:

VCC equipment data and vaccine stock inventories will be stored on a project-specific database on encrypted servers at RBC. Access to the database will be restricted to authorised members of the study team under password-controlled methods as before.

#### Trial oversight

Trial oversight and monitoring will be conducted by the sponsor, the University of Birmingham.

## Discussion

The recent 2024 outbreak occurred just 24 months after the previous outbreak and Mpox will remain an ongoing threat to the health security of low-middle income countries (LMICs). Development of both quantitative laboratory and qualitative point of care immune diagnostics, and implementation of robust vaccination programs where efficacious vaccines exist, are key components of outbreak management.

This study aims to establish Mpox serological testing capability in Rwanda and determine the capacity of the VCC to accommodate additional vaccine deployment. An ELISA is a simple method that is already used in most laboratories and POC LFTs can be used in the field, allowing both to be rolled out to other at-risk countries in the region to support strategies to control and prevent Mpox outbreaks.

As there is no established immune (antibody) correlate of protection, this diagnostic test will be unable to determine if individuals have protective immunity and therefore do not require vaccination. The assay will only measure total antibody to four mpox proteins, rather than functional antibody or T-cell response, which may not provide a full picture of immunity. In addition, there is significant homology between orthopox viruses, leading to cross-reactivity in the immune response. Another limitation of the study is that it will only be conducted in Rwanda, limiting the generalisability to other African nations. Part B of the study relies on manual data entry by staff at different parts of the VCC into a specialised app. To minimise the risk of errors, this app has been adapted from one previously used by staff for an earlier study, and they will be extensively trained.

This study is highly important due to its dual focus on precision diagnostics from a relevant (African) population and operational relevance to the use of vaccines for outbreak control. This study will help to address global health by providing diagnostic tools fit for use and accurate in mpox transmission regions. Simultaneously, quantifying the Rwandan vaccine cold-chain capacity is essential for planning the effective rollout of vaccines while safeguarding existing routine maternal and child health immunisation programs.

## Data Availability

The datasets generated and/or analysed during the current study are not publicly available to protect individual privacy but are available from the corresponding author on reasonable request. All data is owned by RBC who make decisions and manage vaccination strategies.

## Declarations

### Patient and public involvement statement

There was no public involvement in development of the research question or study design. However, the planned semi-structured interviews or focus groups with study participants and healthcare workers will address PPI aspects to the project.

## Abbreviations

CFR: Case fatality rate
DBS: Dried blood spot
DRC: Democratic Republic of the Congo
ELISA: Enzyme linked immunosorbent assay
EPI: Expanded programme of immunization
HIV: Human immunodeficiency virus
hMPXV: Human Monkeypox virus
LFT: Lateral flow test
LMIC: Low-middle income country
NAAT: Nucleic acid amplification testing
NPV: Negative predictive value
OPXV: Orthopoxvirus
PCR: Polymerase chain reaction
PHEIC: Public health emergency of international concern
POC: Point of care
PPV: Positive predictive value
RBC: Rwanda Biomedical Center
RNEC: Rwanda National Ethics Committee
ROC: Receiver operating characteristic
SIB/ICF: Study Information Booklet and Informed Consent Form
SOP: Standard operating procedure
UKHSA: United Kingdom Health Security Agency
VCC: Vaccine cold-chain
WHO: World Health Organization
